# Long-distance migratory shorebirds travel faster towards their breeding grounds, but fly faster post-breeding

**DOI:** 10.1038/s41598-019-45862-0

**Published:** 2019-07-01

**Authors:** Sjoerd Duijns, Alexandra M. Anderson, Yves Aubry, Amanda Dey, Scott A. Flemming, Charles M. Francis, Christian Friis, Cheri Gratto-Trevor, Diana J. Hamilton, Rebecca Holberton, Stephanie Koch, Ann E. McKellar, David Mizrahi, Christy A. Morrissey, Sarah G. Neima, David Newstead, Larry Niles, Erica Nol, Julie Paquet, Jennie Rausch, Lindsay Tudor, Yves Turcotte, Paul A. Smith

**Affiliations:** 1 Department of Biology, Carleton University, Ottawa, ON Canada; 2 Environment and Climate Change Canada, Wildlife Research Division, Ottawa, ON Canada; 3 Environmental and Life Sciences Graduate Program, Trent University, Peterborough, ON Canada; 4 Environment and Climate Change Canada, Canadian Wildlife Service, Quebec, QC Canada; 5 Endangered and Nongame Species, New Jersey Division of Fish and Wildlife, Trenton, USA; 6 Environment and Climate Change Canada, Canadian Wildlife Service, Ottawa, ON Canada; 7 Environment and Climate Change Canada, Canadian Wildlife Service, Toronto, ON Canada; 8 Environment and Climate Change Canada, Science and Technology Branch, Saskatoon, SK Canada; 9 Department of Biology, Mount Allison University, Sackville, NB Canada; 10 Lab of Avian Biology, Department of Biology & Ecology, University of Maine, Orono, ME USA; 11 United States Fish and Wildlife Service, Sudbury, MA USA; 12 Environment and Climate Change Canada, Canadian Wildlife Service, Saskatoon, SK Canada; 13 New Jersey Audubon Society, Bernardsville, NJ USA; 14 Department of Biology and School of Environment and Sustainability, University of Saskatchewan, SK, Canada; 15 Coastal Bend Bays and Estuaries Program (CBBEP), Corpus Christi, TX USA; 16 Wildlife Restoration Partnerships LLC, Greenwich, NJ USA; 17 Department of Biology, Trent University, Peterborough, ON Canada; 18 Environment and Climate Change Canada, Canadian Wildlife Service, Sackville, NB Canada; 19 Environment and Climate Change Canada, Canadian Wildlife Service, Yellowknife, NT Canada; 20 Maine Department of Inland Fisheries and Wildlife, Bangor, ME USA; 21 Département des sciences et techniques biologiques, Collège de La Pocatière, La Pocatière, QC Canada

**Keywords:** Macroecology, Macroecology, Animal migration, Animal migration

## Abstract

Long-distance migrants are assumed to be more time-limited during the pre-breeding season compared to the post-breeding season. Although breeding-related time constraints may be absent post-breeding, additional factors such as predation risk could lead to time constraints that were previously underestimated. By using an automated radio telemetry system, we compared pre- and post-breeding movements of long-distance migrant shorebirds on a continent-wide scale. From 2014 to 2016, we deployed radio transmitters on 1,937 individuals of 4 shorebird species at 13 sites distributed across North America. Following theoretical predictions, all species migrated faster during the pre-breeding season, compared to the post-breeding season. These differences in migration speed between seasons were attributable primarily to longer stopover durations in the post-breeding season. In contrast, and counter to our expectations, all species had higher airspeeds during the post-breeding season, even after accounting for seasonal differences in wind. Arriving at the breeding grounds in good body condition is beneficial for survival and reproductive success and this energetic constraint might explain why airspeeds are not maximised in the pre-breeding season. We show that the higher airspeeds in the post-breeding season precede a wave of avian predators, which could suggest that migrant shorebirds show predation-minimizing behaviour during the post-breeding season. Our results reaffirm the important role of time constraints during northward migration and suggest that both energy and predation-risk constrain migratory behaviour during the post-breeding season.

## Introduction

Every year, billions of animals migrate in search of improved foraging conditions, safety from predators, and enhanced reproductive opportunities^1–3^. Although seasonal migration is widespread among animals, it is especially well studied in birds^4^. During birds’ pre-breeding migration (i.e., the boreal spring for temperate and Neotropical migrants), it is generally assumed that individuals are more time constrained in reaching their goals than during post-breeding (boreal autumn) migration. This is because of: (i) competition among individuals where the timing of arrival provides advantages in acquiring the best breeding territories^5^, (ii) the importance of matching temporally narrow peaks in food abundance^6^, and/or (iii) for Arctic breeding birds, the need to breed early to maximize re-nesting opportunities given the compressed breeding season^7^.

Because of these different time constraints, theory predicts that migratory birds should adopt a time-minimization strategy in the pre-breeding season^8^. Birds employing this strategy should devote as much energy as possible to fuel deposition and consequently avoid other time and energy consuming activities such as moult on their stopover site, leading to a faster migration^8,9^. During the post-breeding season however, when time constraints are less, migrants should shift towards an energy-minimizing strategy, i.e., to reduce their energy expenditure as much as possible^10^, and to recover their energy reserves after an energetically demanding breeding season. Birds following this strategy are predicted to stop more *en route* to refuel^10^ and because they are less time constrained^11^, they may be more inclined to wait for favorable migration conditions^11^.

Many empirical studies have supported elements of these general predictions relating to pre-breeding versus post-breeding migration^12–14^. However, not all studies have detected seasonal differences in migration speed^15–17^, and several studies have found patterns opposite to the above predictions^18–21^. These reverse patterns suggest that there may be some advantage for a slower migration in the pre-breeding season, such as to minimise energy expenditure prior to breeding, or it could result from atypical environmental conditions such as cold temperatures or high ice cover at breeding areas that slow down this pre-breeding migration. These environmental conditions are potentially unpredictable in space and time, possibly confounding general patterns. For example, the optimal speed to achieve time- or energy minimization depends on the rate of energy acquisition at current and future stopover sites, and the rate of migratory progress depends on the head- or tailwinds encountered^8^. Environmental variables can therefore have a strong influence on the rate of migratory progress^22^, and because these environmental factors can be difficult or impossible to predict^23^, birds’ migration strategies must also incorporate elements of risk avoidance^24^.

In addition to these weather related risks, another important risk that could influence migration behaviour is the risk of predation^25–27^. High departure fuel loads and changes in organ size required by time-minimizing shorebirds impair take-off performance and manoeuverability, compromising their ability to escape from predators^28–31^. Migratory shorebirds forage in dense flocks, and because of their dependence on intertidal mudflats to forage, they congregate in large numbers on only a few sites along their migratory route to refuel for migration north and south^32^, thereby attracting a variety of avian predators^33,34^.

Raptors, especially peregrine falcon *Falco peregrinus*, merlin *Falco columbarius* and Cooper’s hawk *Accipiter cooperii* are key predators of migrant shorebirds in the Atlantic flyway of North America^33,35^. During the pre-breeding season, these raptor species migrate north over a protracted period and initiate migration before most shorebirds, as they travel to their breeding grounds in the vast tundra and boreal regions of North America; breeding areas which are at lower latitudes than for arctic-breeding shorebirds^36–38^. In contrast, after the breeding season, these raptors migrate during a narrower time window and concentrate in space and time as they head south, due to the inverted triangular shape of the continent^38,39^. Therefore, migratory shorebirds face a greater risk from migratory raptors during the post-breeding season versus the pre-breeding season^40^. Most migrant shorebirds begin their southbound migration ahead of the wave of migrating raptors; thus, a slow post-breeding migration for shorebirds could increase the overlap with these avian predators^27^.

Identifying seasonal differences in migratory decisions and performance is crucial for understanding the ecological and evolutionary constraints that shape migratory behaviour^12,41^. To identify seasonal differences in the speed of migration, most studies have used overall migration speed (km day^−1^) as the behavioral metric^12,14^; a metric which describes the northward or southward progress including stopover time *en route*, and given the environmental conditions experienced. However, seasonal differences in these environmental conditions, specifically head- or tailwinds, can profoundly impact migration speed even without any change in birds’ behaviour; therefore, it is essential to incorporate other behavioural and environmental (wind) metrics in assessments of birds’ migration behaviour. For example airspeed, a bird’s flight speed relative to the surrounding air, might better indicate whether birds are more time constrained in the pre-breeding season versus the post-breeding season. Airspeed more directly reflects the birds’ flight behaviour and the energy costs incurred by that behaviour, after accounting for any seasonal differences in wind that could influence total migration duration^42,43^.

Here, we test the hypothesis that on the North American continent, Arctic-breeding shorebirds (extreme long-distance migrants) display relatively more time-minimizing migration behaviour pre-breeding, and energy-minimizing behaviour post-breeding. Since there is evidence for age-specific differences in migration speed^44,45^, we also explore potential age differences. We predict that migration speed and the airspeed of shorebirds is faster during the pre-breeding season compared to the post-breeding season. Because the rate of energy expenditure during migratory flights is higher than the refuelling rate, most of the migration period is spent at stopover sites^8,10^, suggesting that stopover duration is the main driver of migration speed^14^. Pre-breeding migrants are therefore expected to stop less often and for shorter periods *en route*, and move more rapidly towards their breeding grounds. After the breeding season, because energy minimizers are inclined to wait for favorable migration conditions^11^, we predict that birds will be more wind selective, less goal oriented, and have a longer migration period. However, this protracted migration could expose birds to a “wave” of migrant predators^39^. Therefore, we also explore the evidence for predation-minimizing behaviours^27^. More specifically, we evaluate shorebird migration speed in relation to the timing and abundance of avian predators at key raptor migration sites. To evaluate these predictions, we used automated VHF telemetry on a continental scale^46^ and followed the seasonal migration of Arctic-breeding red knots *Calidris canutus rufa*, sanderlings *C. alba*, semipalmated sandpipers *C. pusilla* and ruddy turnstones *Arenaria interpres* over multiple years. The fine temporal resolution of these automated telemetry data allow us to explore the migration speed, airspeed and timing of departure in a way that was not previously possible on this temporal and geographic scale. The results provide new insights into the factors underlying migratory strategies for these extreme long-distance migrants.

## Results

**Figure 1:**
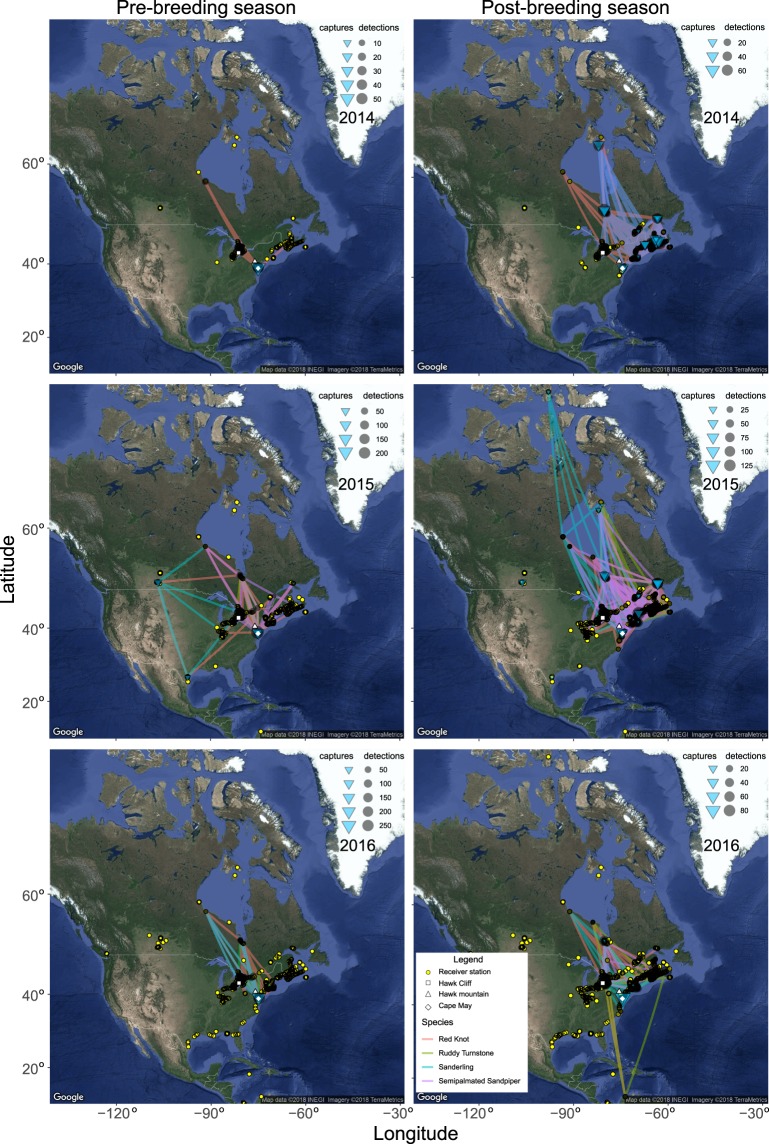
Locations of active receiver stations (yellow dots with black outlines), and the migratory trajectories of the birds used in analyses. The lines (great circle routes) connect detections for individuals of each of the four shorebird species (tracks are coloured per species; Red Knot = pink, Ruddy Turnstone = green, Sanderling = turquoise and Semipalmated Sandpiper = purple), with panels separating year and season. These tracks represent simplified flight trajectories; birds may have deviated from these great circle routes. Maps created using R 3.4.3 using packages ggplot2^84^ and ggmap^85^ (image data providers: US Dept. of State Geographer© 2018 Google).

A total of 1,937 shorebirds were fitted with a transmitter between 2014 and 2016 (see Fig. 1 and Table S1 for details); 647 during the pre-breeding and 1,290 during the post-breeding season. Of these, we were able to calculate the total migration speed of 516 individuals and airspeed for 389 individuals that were detected at least twice > 50 km apart within the same season. Among the remainder, 88 (< 5%) individuals were not detected anywhere in the network indicating the tags may have malfunctioned, fallen off, or otherwise escaped detection; 804 were only ever detected at a single receiver station, usually near the tagging location; 362 individuals were detected at multiple towers but < 50 km apart, which we considered local movements and did not include in further analyses; and 255 individuals were detected at greater distances in different seasons, but only at one station within each season, thus preventing use in analyses.

### Migration speed, stopover and predator avoidance

**Figure 2:**
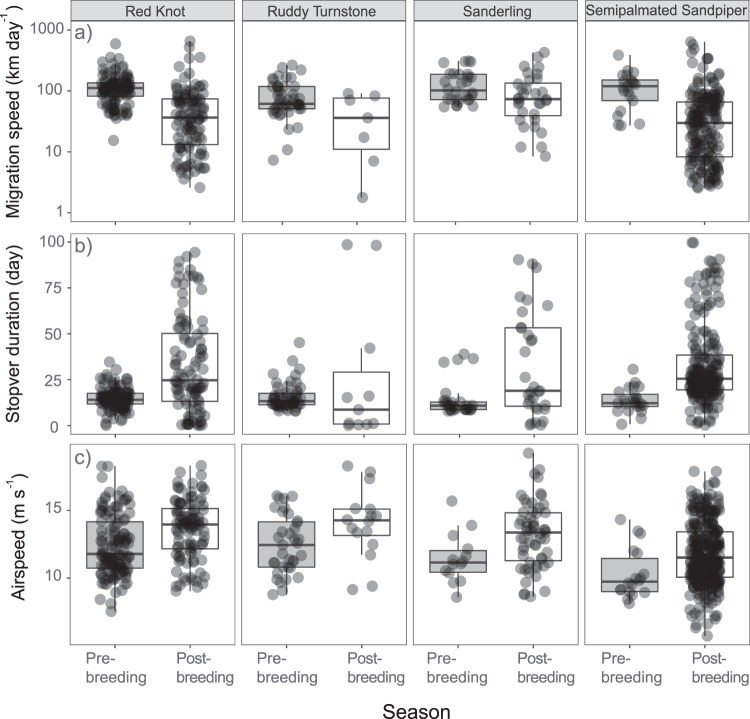
Model estimates of (**a**) total migration speed per day, (**b**) minimum stopover duration and (**c**) airspeed of four species of Arctic-breeding shorebirds during pre- and post-breeding migration. Estimates are derived from linear mixed models (see text) and the box-and-whisker plots give median (horizontal line within box), interquartile range (box), range (bars) and the transparent dots show estimates for individual birds.

As predicted, migration speed (km day^−1^) prior to the breeding season was more rapid than after the breeding season for all four species considered (GLMM, *p* < 0.001; Fig. 2a). The final model for migration speed included season (*X*² = 17.4, *p* < 0.001) and species (*X*² = 75.2, *p* < 0.001), along with random effects of year and capture site. Northbound transit was almost twice as fast across species versus the southbound transit (116 km day^−1^ during the pre-breeding season versus 62 km day^−1^ post breeding). In accordance with predictions, based on our final model which included season, all species stopped for longer periods during post-breeding migration (GLMM, *X*² = 73.0, *p* < 0.001; Fig. 2b). The mean minimum stopover duration (i.e., sum of all stopover duration per season), for pre-breeding migrants was 14.7 ± 0.4 d (SE) across species, whereas for post-breeding individuals the mean stopover duration was 32.6 ± 1.3 d (SE).

In contrast to these patterns, the airspeeds were ~10% higher during the post-breeding season for all species (GLMM, *X*² = 6.1, *p* = 0.01; Fig. 2c), suggesting that birds had higher levels of energy expenditure during the southbound migration flights. These higher post-breeding airspeeds correspond to ~10% increase in instantaneous flight cost (W kg^−1^), based on power curve calculations (see Table S2 for details).

**Figure 3:**
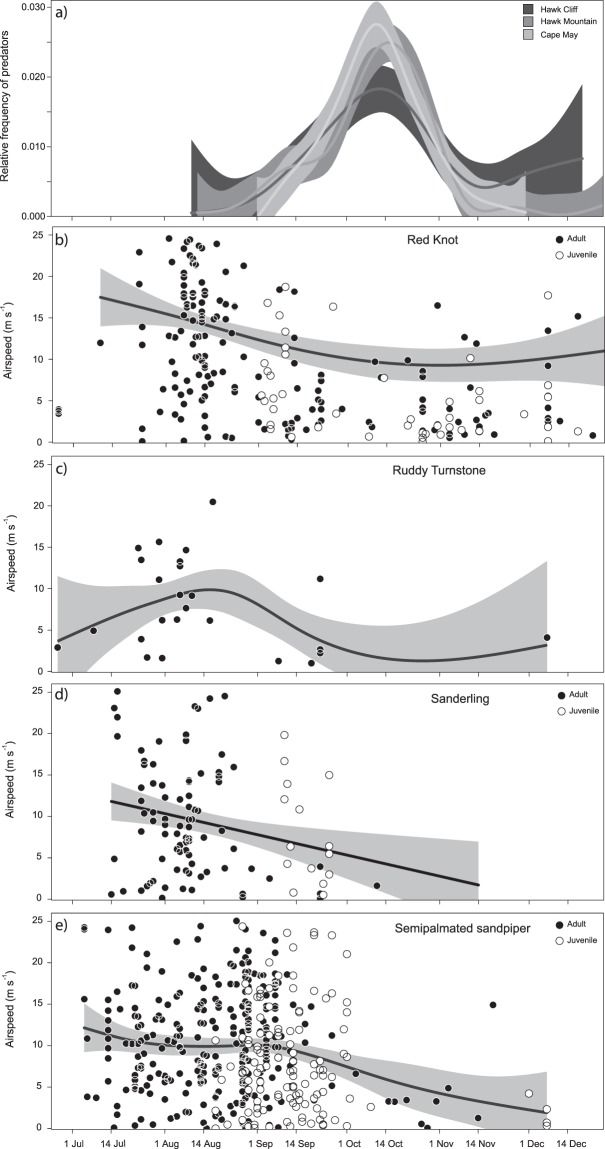
Mean relative frequency of avian predators at three sites in North America (see text for details) and airspeeds of 4 shorebird species separated by age class, throughout the protracted post-breeding migration period. The airspeeds are higher prior to the wave of predators. The solid line represents the regression (linear model or GAM; see text) and the grey area indicates the 95% confidence intervals of the regression.

Predator abundance showed a clear and simultaneous seasonal peak after the breeding season at three key raptor migration sites (GAM_Hawk Cliff_, *F* = 7.9, *R*^2^ = 0.32; GAM_Hawk Mountain_, *F* = 28.8, *R*^2^ = 0.65 and GAM_Cape May_, *F* = 26.8, *R*^2^ = 0.69; Fig. 3a). All shorebird species showed similarities in their seasonal patterns of airspeed (GAM_red knot_, *F* = 19.2, *R*^2^ = 0.33, Fig. 3b; GAM_ruddy turnstone_, *F* = 2.9, *R*^2^ = 0.18, Fig. 3c; LM_sanderling_, *F*_*1,105*_ = 8.1, *R*^2^ = 0.08, Fig. 3d; GAM_semipalmated sandpiper_, *F* = 4.8, *R*^2^ = 0.05; Fig. 3e), with peaks in airspeeds prior to the peak of raptors and reduced airspeeds after the peak occurred (GLMM, *X*² = 3.7, *p* = 0.024).

### Timing of migration

**Figure 4:**
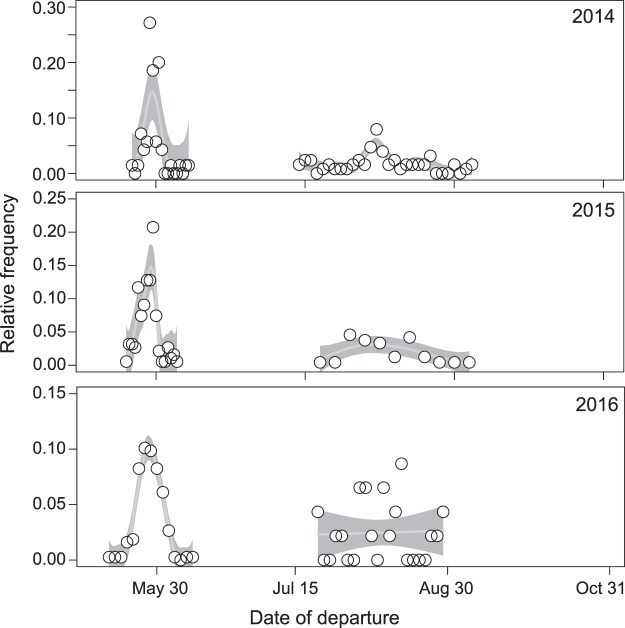
Timing of migration for the four species combined, as determined from individual departure dates from Delaware Bay (pre-breeding) and the southern James Bay region (post-breeding), separated by year. The white dots represent the relative frequency of individuals departing the sites and the lines and shaded areas represent estimated model means and 95% confidence intervals. The timing is highly synchronized during the pre-breeding season, but not during post-breeding.

Pre-breeding departure timing was highly synchronized across years and showed a narrow time window, whereas in the post-breeding season the departures were distributed over a longer period (Fig. 4). Large numbers of shorebirds stage in Delaware Bay (New Jersey and Delaware, USA) before making their final non-stop flight into their Arctic breeding grounds^47^. After the breeding season, the Southern James Bay region (Canada, ON), is one of the key stopover sites on the Southern edge of the Arctic breeding sites^48^. The mean date of departure from Delaware Bay was (day of year) 148.4 ± 3.5 days (mean ± SD; 148 = 28 May), and all species departed close to this date (day of year 148.2 ± 2.9 days for red knots; 148.9 ± 3.8 days for ruddy turnstones; 150.9 ± 3.8 days for sanderlings and 146.6 ± 9.8 days for semipalmated sandpipers). The mean date of departure from Southern James Bay during the post-breeding season for adults was 231.9 ± 15.6 days (mean ± SD, 232 = 20 Aug). These departure dates did not vary significantly across species (GLMM, *X*^2^ = 1.6, *p* = 0.64), but differed between adults and juveniles (GLMM, *X*^2^ = 52.2, *p* < 0.004), with juveniles leaving about 19 days later.

### Wind support and flight directions

**Figure 5:**
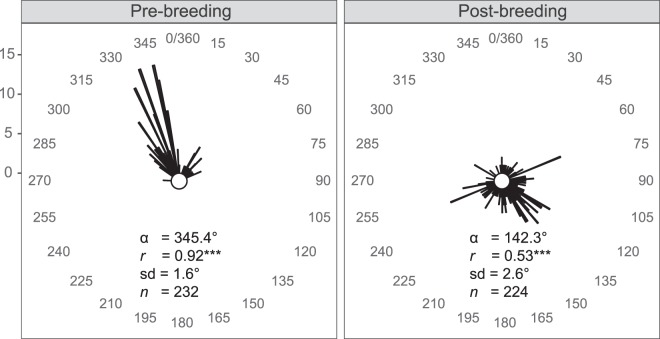
Circular distributions of migratory flight bearings (in degrees) for the four shorebird species and three years combined, for pre- and post-breeding migrations. Each line shows the direction of tracked individuals and the length refers to the number of movements in a given direction (scale 0–15). Each line is a measurement of a migration segment, which may represent only a part of the detected migratory route and therefore includes some non-independent measurements across individuals. The inset shows (α) the mean track direction, (*r*) vector length, (sd) angular deviation and (*n*) sample size per season. The statistical significance refers to a Rayleigh test (***p < 0.001).

Most individuals showed a high degree of wind selectivity in both seasons. Wind support on the observed departure date was higher than the wind support on the preceeding 10 days (GLM, *X*² = 100.6, *p* < 0.001; see Supplementary Fig. S1 for details). The relative wind support at the time of departure was greater for the post-breeding versus pre-breeding migration (GLM, *X*² = 445.0, *p* < 0.001); birds experienced more supportive tailwinds during the post-breeding season. However this greater wind support does not account for the differences in birds’ airspeeds between seasons, as airspeed accounts for head- or tailwinds (i.e., represents the flight speed in still air). A wider range of flight directions were utilized post-breeding (as indicated by a lower vector length (*r*); Fig. 5), suggesting a less clear orientation towards a destination, which is consistent with an energy minimizing strategy. No difference in orientation between the age classes was observed (*p* < 0.10, Watson’s *U*^2^ test).

## Discussion

Our result of a faster migratory progress prior to breeding is consistent with the basic predictions of optimal migration. The difference in migration speed between both seasons appeared to reflect differences in stopover duration. Our results indicate that the airspeed of shorebirds during the pre-breeding season is not at its upper limit, as the estimated pre-breeding airspeeds were below those post-breeding, and also below the theoretical maximum-range speed (*V*_mr_; Table S3). This is surprising given the wide acceptance that birds respond to the time constraints imposed by the breeding season by minimizing the duration of the pre-breeding migration. Our findings suggest that other considerations such as body condition upon departure^41,49^ or upon arrival at the breeding sites^50^, may influence a bird’s migratory decisions during the pre-breeding season to a greater degree than previously believed.

An increase in airspeed comes at a cost of increased flight power, which necessitates increased fueling. The optimal airspeed for maximizing total migration speed (or equivalently, minimizing the total time of migration) is 5–15% higher than the optimal airspeed for minimizing energy costs per unit distance^8,51^, leading to an increase in total migration speed of only 0.2–2%^42,52^. Therefore, increasing airspeed to increase the speed of migration might be disadvantageous during the pre-breeding season, when body condition offers other benefits upon arrival to the breeding grounds. Conversely, post-breeding and juvenile birds during the post-breeding season face a different suite of temporal and energetic constraints, and importantly, are faced with increased predation pressure due to potential co-occurrence with migrating raptors.

Shorebirds are primarily income breeders (i.e., they acquire all the necessary resources on the breeding grounds)^53^; however, body condition upon arrival to the breeding grounds can nevertheless play an important role in the survival and reproduction of Arctic-breeding shorebirds. Several studies have demonstrated that individuals arriving to the breeding grounds with greater body stores are able to withstand harsh early-season weather^50^, and high body condition during the pre-breeding period has been linked to higher quality eggs^54^. Red knots with greater energetic reserves during spring migration remained in the Arctic longer and were more likely to be detected after the breeding season, suggestive of successful breeding and greater survival, respectively^41^. Maximizing airspeeds during the pre-breeding season could impose a significant cost of reduced body stores upon arrival but yield only a modest benefit in terms of reducing the total duration of the migration.

Surprisingly, we found only minor age-related differences in migratory behaviour, such as timing of migration for red knots, sanderling and semipalmated sandpiper (Fig. 3b,d,e; we did not have juvenile ruddy turnstone in the data). Our post-breeding migration data were collected at Arctic, subarctic and north-temperate latitudes close to the breeding sites (in some cases, departing from the breeding sites). Age-related differences in migration behaviour might occur later in the post-breeding season, farther south. Although some shorebirds are known to migrate in groups of variable age classes^55^, others show more distinct waves of adults and juveniles^56^. Nevertheless, inexperienced juveniles may benefit from social information for navigation and stopover site use during their first post-breeding migration.

Predation risk is a factor affecting birds’ migratory behavior in a profound way^25,33^. Although direct mortality from predation can be significant, non-lethal effects can illicit variable behavioural responses in a wide range of taxa^57,58^, including in shorebirds^25,59^. Among shorebirds, responses to increased predation danger include behavioural changes such as variable stopover duration^33^, habitat use^9^, body mass changes^60^ and even morphological changes^48^. Here we showed that post-breeding birds that migrated in advance of the “predator front”, had higher airspeeds. Later in the season, after the peak of predation risk had passed, airspeeds were lower and similar to those observed during pre-breeding. This observation is consistent with the mortality-minimizing hypothesis, which predicts that when the predation front is approaching, individuals should increase the fuel-loading rate to increase migration speed^27^.

Wind is another factor with a crucial influence on the speed of migration; headwinds or tailwinds directly reduce or increase the speed of migration for a given bird’s airspeed. However, wind can also alter the decisions made by migrants. An individual that is maximising the migration speed should increase its airspeed in headwinds and decrease its airspeed with tailwinds^61^. As we have shown, the different shorebird species are highly wind selective and predominantly initiate migration on days with supportive tailwinds. However, our results show that birds migrate with higher tailwinds in the post-breeding season (Fig. S2), suggesting that they should lower their airspeed to maximise flight range. Therefore, the higher airspeeds in the post-breeding season are not the result of migrating into headwinds.

During the post-breeding season, birds also exhibited a high flexibility in their departure direction to capture these tailwinds, as shown by the wider range of post-breeding departure bearings. Together, these results of high airspeed, longer stopover durations and flexible departure directions suggest the possibility that post-breeding migration is influenced by the constraints of predation risk, despite the fact that post-breeding migration speed is lower compared to the pre-breeding season. Increased predation risk post-breeding could potentially reduce fueling rates due to increased vigilance^62^, which could in turn lead to a lower departure mass from a stopover site. This low departure mass coupled with the high airspeed observed in this study could increase the required stopover duration(s) further south in the migration. Alternatively, the urge to depart from sites where predators were encountered is greater than the drive to fly in a specific direction. Competition, deteriorating weather^63^ and declining food availability might also drive shorebirds to move out of the area^64^ and temporarily speed up their pace, forcing them to stop more and/or longer subsequently *en route*.

For most Arctic-breeding shorebirds, the post-breeding season also brings on moult; species show a great variability in timing of moult as they may moult prior to, during, or after southward migration^26^. Missing feathers will cause a reduction of wing area and/or wing span, and flight costs are altered during active moult. However, few species undertake active moult during migration (e.g., dunlin *Calidris alpina*^65^), and the energetic effects of moult on flight performance are generally small^66^. Although variation in moult across the species and individuals studied here could increase the observed variation in airspeed, it cannot explain faster airspeeds in the post-breeding season.

Our finding of a higher airspeed for migratory shorebirds during post-breeding has not previously been documented, likely in part because of the difficulties of measuring flight speeds for small-bodied migrant birds. Until recently, tracking technologies for small-bodied migrant birds have not offered both large-spatial coverage for tracking long-distance migrations and fine temporal resolution for understanding these subtle changes in behaviour. However, our automated telemetry array does not cover the full length of the flyway. Birds in our dataset might alter their behaviour after they leave our study areas. Possibly, northbound individuals initiate a final sprint to their breeding sites and counterbalance the mean differences in airspeed between both seasons over the entire trajectory^67^. Likewise, potential differences in migratory behaviour such as protandry (males arriving to breeding areas before females), which is common in many bird species^68^, cannot be excluded, as unfortunately, we did not have sufficient data concerning the sexes of the species involved. Although we tracked some individuals to the southern edge of the breeding grounds, most species migrated further north, where these intersexual differences may have been more pronounced. Nevertheless automated telemetry results such as these, with fine temporal resolution, offer fertile ground for explorations of the predictions of optimal migration theory. Location-specific counts of predators, and location-specific assessments of departure behaviour, might allow for a further refinement of the understanding of the role of predators in shaping the migration behaviour of long-distance migrant birds.

## Methods

As part of several ongoing studies of shorebirds in North America, red knots, sanderlings, semipalmated sandpipers and ruddy turnstones were captured between 2014 and 2016 and measured using standard protocols at 13 locations distributed widely across the Atlantic flyway from Texas, United States, to Nunavut, Canada (see Fig. 1 and Table S1 for details). All birds were banded with numbered metal bands, and standard biometric measurements were taken. Age was determined from species-specific plumage features and classified as hatch-year (first post-breeding migration) or after-hatch-year (experienced at least one previous southward migration), while sex could not be determined at capture based on morphology^69^. Immediately after these measurements were taken, a sample of birds were fitted with digitally coded radio-transmitters (Avian Nanotags, Lotek Wireless Inc., Newmarket, ON, Canada), which have an estimated life span of 33–150 days, depending on type and burst rate (see below). The radio tags were glued to the skin and clipped feather stubble of the synsacral region with a cyanoacrylate gel adhesive. Tag transmissions were tracked using a network of automated radio telemetry receiving stations, the Motus Wildlife Tracking System^46^, with towers distributed across North America and to a lesser extent in South America.

Receiver stations were equipped with a digital telemetry receiver (Lotek SRX600, Lotek SRX800, or a SensorGnome receiver; www.sensorgnome.org), and most receiver stations had multiple directional antennas oriented at fixed angles (typically three to six 9-element Yagi antennas distributed evenly around 360°) that scanned continuously for tags. All tags operated on a single frequency (166.380 MHz) and were distinguished by a unique series of pulses contained within each burst of radio transmission (burst rate: 5.3–14.9 s), allowing for definitive identification of individuals. Tagged birds can be detected at distances up to 20 km away from a receiver station, although topography, weather, and vegetation can all decrease detection range^46,70^. Tag detections were recorded by the automated receivers and time stamped by an internal GPS clock, allowing us to track individual movements at a continental scale with a temporal resolution of ±15 s.

### Analysis of automated telemetry data

We processed the data as previously described^41,71^. In summary, all tag, station, project, and user metadata are submitted by users, archived in the database, and linked and managed through the Motus research platform^46^. The tag detection data collected at receiving stations are joined with the master tag and station metadata to produce a complete database of unique detections from each station. The radio signals captured by the receivers are cross-referenced against the tag recordings submitted to Motus during tag registration. Due to local interference and the high gain on the receivers, spurious signals (i.e., false positives) were recorded. From the raw detection data, detections that are outside of the deployment period for a specific tag were removed. We removed detection data where the standard deviation in signal strength was > 0.1 and the run length < 2 (number of continuous detections of a unique code by a receiver). Detections in this filtered database (containing tag-id, tower location, GPS time stamp, antenna number, and signal strength), were considered valid if there were three detections from a tag at multiples of the tag’s burst rate (±4 ms, with the allowed error in the burst rate increasing by 1.5 ms for each missed burst and allowing for up to 20 consecutive missed bursts). Although this filtering procedure might remove some valid detections, it ensured that the resulting data set contained only valid detections of the tagged birds.

Departure time (UTC) from each site was determined as the last of at least three consecutive detections prior to no further detections at a site. Arrival times (UTC) were determined as the first detection in a series of at least three consecutive detections at a receiver station within a different site.

Ground speed (m s^−1^) was calculated as the orthodrome distance (m) between two different receiver stations, divided by the total time (s) between departure and arrival. As the objective was to determine migration speed, we excluded local movements (< 50 km). The mean distance between all detections was 550 km and therefore the bias from the uncertainty of an individuals’ actual position (e.g., a 20 km detection distance), is likely not a large factor influencing the speed calculations.

We calculated the minimum stopover duration for each individual as the sum of all time differences (s) between the last and first detection per receiver station, including the time an individual remained at the capture site after capture. A stopover event was defined as a minimum duration of 30 min, and shorter durations were considered migratory movements^41^.

Migration speed (km day^−1^) was calculated as the sum of the orthodrome distance (km) between all receiver stations per season at which an individual was detected, divided by the total time an individual was detected, including the minimal stopover time.

When an individual had been detected between two receiving stations, we calculated the bearing in degrees using the package fossil^72^ in the program R^73^. We only used the bearings describing departures from one key site per season; Delaware Bay for pre-breeding migration (39°4′N and 74°6′W) and the southern James Bay region for the post-breeding migration (51°4′N, 80°4′W). A majority of birds were detected at these sites, and they are of known importance for these species^47,48^. Analysing bearings in this way minimizes the potential bias arising from site-dependent departure directions, and the fact that northbound migrants in spring are heading towards higher latitudes where there are fewer towers (see Fig. 1). Based on these bearings, we calculated the mean vector length *r* (which can vary from 0 to 1) as a measure of directional concentration, and the Watson *U*^2^-test was used to test whether the age classes differed from each other.

### Wind data

We used the wind data of the National Centers for Environmental Prediction (NCEP). The flow-assistance experienced by each individual was calculated using the R-Package RNCEP^74^. Since we recorded when and where an individual is present and the destination (i.e., different receiver station) is known, we used the latitudes and longitudes of the receiver stations as the start- and end-point for each trajectory, with the corresponding departure times. Using this information, we downloaded the –u (west-east) and –v (south-north) wind components, which were combined in a single wind vector incorporating the strength and the direction of the wind, from which we obtained a tailwind component. The calculations were based on wind components for the following pressure levels: ‘surface level’, 925, 850 and 700 hPa, corresponding to between 0 and 3000 m altitude. The RNCEP tailwind model was set to calculate the heading and tailwind component at the most optimal pressure level upon departure, and this pressure level was reassessed every 3 hr, and the heading and tailwind component recalculated by the model. In this way, we obtained an estimate of the wind assistance experienced by birds every 3 hr, assuming that they travelled at the most profitable pressure level.

Airspeed (m s^−1^) was calculated by subtraction of the wind vector (wind assistance) from the track vector (ground speed, see above) to estimate individual airspeed^75^. Some track segments (< 3% of total sample) had airspeeds > 25 m s^−1^, which were the result of occasional false detections of tags (e.g., near simultaneous apparent detections of a bird at towers hundreds of kilometers apart). These false detection patterns were identified by examining plots of detections for each bird by latitude and time and longitude and time, and values associated with unrealistic movement patterns were removed^76^. Airspeeds below a threshold of < 5 m s^−1^ were considered as possible undetected stopovers or detours through areas with no tower coverage^77^, and were excluded from airspeed analyses. Our power curve calculations (see below) indicate that the power required to fly below these speeds increases exponentially making these speeds unlikely. In order to identify the costs associated with airspeeds, we calculated the power curves for the four species using the program Flight 1.24, which is available (free) from http://books.elsevier.com/companions/9780123742995 (see Tables S2 and S3 for details).

In order to evaluate the hypothesis that long-distance migratory shorebirds would be more wind selective in the post-breeding season, we calculated wind support for each individual at the actual departure time and location, and calculated the wind support for the same departure time for the period up to 10 days earlier.

### Predator density data

We accessed the citizen science data of the Hawk Migration Association of North America^78^, and selected three important raptor post-breeding migration count sites in North America; Hawk Cliff Hawkwatch site in Ontario, Canada (42°39′N, 81°10′W), Hawk Mountain Sanctuary in Pennsylvania, USA (40°38′N, 75°59′W) and Cape May Point, New Jersey, USA (38°55′N, 74°57′W; see Fig. 1). Experienced volunteers enter counts of migrating raptors on a daily basis. We obtained daily observations from 2014–2016, for the period between early July and late December. Peregrine falcon, merlin and Cooper’s hawk were selected as relevant predators for the four shorebird species considered here. We corrected the counts for the daily observation duration, generating a daily mean predator rate (number hr^−1^). Next, we used mean values of the three years and calculated the relative frequency per day.

### Statistical analysis

Differences in migration speed (km day^−1^) between seasons were examined using a generalized linear mixed model (GLMM)^79^. The model included migration speed (km day^−1^) as the predicted variable with year and capture site as random factors. We started with a full model including season, average tailwind along all segments, age, species and all possible interactions, and then simplified the model using a backwards elimination process based on a log-likelihood ratio test (LRT) with *P* < 0.05 as the selection criterion (“drop1” in R) until reaching the minimal adequate model. Unexpectedly, age and tailwind were not included in the final model.

To determine differences in airspeed between seasons, airspeed was calculated only for the period when individuals were flying (i.e., stopover time was not included). Because some individuals were detected along several segments of the journey, they reoccur in the analysis. Therefore, we used a GLMM with airspeed as the predicted variable, and year, capture site and bird-id as random factors. The predictor variables were season, species, age, days after capture and all possible interactions and we simplified the model based on a log-likelihood ratio test (LRT). The final model included season and species. In order to identify whether airspeeds differed prior to and after the predator front, we selected two periods in the post-breeding season based on a visual inspection of the predator front (Fig. 3a); early (> 29 July & < 1 Sept) and late (> 31 Aug). We used a GLMM with airspeed as the predicted variable, and year, capture site and bird-id as random factors. The predictor variables were season, species, age and all possible interactions and simplified the model based on a LRT. The final model included season, species and the interaction term. In order to visualize these patterns, we used generalized additive models (GAM) to describe patterns of predator abundance and airspeed during the post-breeding season and used separate models for each species. The response variable for these models was either relative predator frequency or airspeed. For both models we used day of year as an explanatory variable. The models were fitted using the ‘mgcv’ package^80^ in R ver. 3.4.1, and compared with linear models (LM) based on the Akaike information criterion, and the final model was considered to be substantially better when its value was at least two AIC units lower than the next best model^81^.

Minimum stopover duration was analysed in the following way: the full model included minimum stopover duration (d) as the response variable with year and capture site as random factors, and season, average tailwind, age, species and all possible interactions as covariates. We then simplified the model based on a LRT until we reached a minimal adequate model.

We calculated the relative proportion of birds departing daily from Delaware Bay during the pre-breeding season and from the southern James Bay region in the post-breeding season to assess the total time of the migration as a measure of time constraints. We used adults only, as age classes are known to differ in timing at our study sites^82^, and these sites were chosen because most birds were detected at these sites.

In order to investigate whether wind selectivity differed between the seasons, wind support for each segment was analysed using LRT of GLMM’s with year and bird-id as random factors and species and season as fixed factors. We used the flow-assistance from NCEP, (see above) to calculate wind support for each migratory trajectory up to 10 days before actual departure.

Prior to all analyses, explanatory variables were assessed for collinearity using the variance inflation factor (VIF) function. All variables had a VIF < 2 and coefficients did not switch between positive and negative values, indicating low multicollinearity^83^.

### Animal handling and ethics

The research was conducted in accordance with the Animal Welfare Act of 1970 and the most recent revision of the Ornithological Council’s guidelines in the use of wild birds in research, and by the Institutional Animal Care and Use Committee (IACUC). Birds captured in Delaware Bay fell under the federal banding permit issued to the Endangered and Nongame Species Program, Division of Fish and Wildlife, NJ DEP (22803). Captures in New Jersey were done under federal banding permit (21241US), issued to New Jersey Audubon by the United States Geological Service/Bird Banding Laboratory. Individuals captured in Quebec fell under the protocol approved by the “Comité institutionnel de protection des animaux” under permit 2011-M29-2 and 2015-M29-1, and the capture of individuals in the Mingan Archipelago was approved by the Animal Care Committee of Environment and Climate Change Canada (SCF2018-01-YA, SCFQ2017-06, SCFQ2016-07). For Sanderling and Red knots captured in Chaplin, Canada, all animal handling and research protocols were approved by the University of Saskatchewan Animal Care Committee (AUP 20120021) and conducted under Canadian Wildlife Service Scientific Permit (12SKS009). Birds trapped in Texas, fell under the Texas Parks & Wildlife Department Permit (SPR-0911-341), and trapping on the Padre Island National Seashore was approved by the National Park Service under permits 2015-SCI-0006, 2015-SCI-0007, and 2016-SCI-0005. All activities related to bird capture and tagging in Maine were reviewed and approved by University of Maine IACUC (A2013-01-02) and were performed under federal and state permits to the U.S. Fish & Wildlife Service and the Maine Department of Inland Fisheries and Wildlife. At James Bay, birds were banded under a permit from Environment and Climate Change Canada (Permit 10884), and banding and tagging was approved by Environment and Climate Change Canada’s Wildlife Eastern Animal Care Committee (protocol 14CF01, 15CF01, 16CF01 and 17CF01), as well as by Trent University’s Animal Care Committee (23904). Birds captured in Nunavut were approved under the Canadian Wildlife Service Scientific Permit to Capture and Band Migratory Birds (10565M), the Canadian Wildlife Service National Wildlife Area Permit (NUN-NWA-14-03), the Canadian Wildlife Service Scientific Research Permit (NWT-SCI-13-01), the Government of Nunavut Wildlife Research Permit (WL2015-032), and by Environment and Climate Change Canada Western and Northern Animal Care Committee Approval (15JR01). Birds captured and tagged at the Bay of Fundy were approved under Mount Allison University Animal Care protocol (12–10).

## Supplementary information

Supplementary Materials

## Data Availability

All data are available by request through the Motus Wildlife Tracking System (www.motus.org).
