# Supplementary material for: Long-distance migratory shorebirds travel faster towards their breeding grounds, but fly faster post-breeding
Source: Sci Rep. 2019 Jul 1;9:9420. doi: 10.1038/s41598-019-45862-0 (PMC6603026; doi:10.1038/s41598-019-45862-0)
Supplement: Supplementary file 1 — Supplementary Materials [file 41598_2019_45862_MOESM1_ESM.docx]

Online Electronic Supplementary Information for:

**Long-distance migratory shorebirds travel faster towards their breeding grounds, but fly faster post-breeding**

Sjoerd Duijns, Alexandra M. Anderson, Yves Aubry, Amanda Dey, Scott A. Flemming, Charles M. Francis, Christian Friis, Cheri Gratto-Trevor, Diana Hamilton, Rebecca Holberton, Stephanie Koch, Ann E. McKellar, David Mizrahi, Christy A. Morrissey, Sarah Neima, David Newstead, Larry Niles, Erica Nol, Julie Paquet, Jennie Rausch, Lindsay Tudor, Yves Turcotte & Paul A. Smith

**Table S1.** List of individuals with season and locations of deployed tags.

|  |  |  |  |  |  |  |  |  |  |
| --- | --- | --- | --- | --- | --- | --- | --- | --- | --- |
|  |  |  |  |  |  |  |  |  |  |
| Year | Season | Common name | Scientific name | Capture site | Lat. | Lon. | Ind. tagged | |  |
|  |  |  |  |  |  |  | Adult | Juvenile |  |
|  |  |  |  |  |  |  |  |  |  |
| 2014 | Pre-breeding | red knot | *Calidris canutus* | Delaware Bay, NJ,USA | 39 | -75 | 86 | 0 |  |
| 2014 | Post-breeding | red knot | *Calidris canutus* | Cape Cod, MA, USA | 42 | -70 | 1 | 1 |  |
| 2014 | Post-breeding | red knot | *Calidris canutus* | Mingan Archipelago, QC, Canada | 50 | -64 | 0 | 16 |  |
| 2014 | Post-breeding | red knot | *Calidris canutus* | Delaware Bay, NJ, USA | 39 | -75 | 8 | 0 |  |
| 2014 | Post-breeding | red knot | *Calidris canutus* | James Bay south, ON, Canada | 51 | -80 | 1 | 0 |  |
| 2014 | Post-breeding | red knot | *Calidris canutus* | Monomoy, MA, USA | 42 | -70 | 14 | 5 |  |
| 2015 | Pre-breeding | red knot | *Calidris canutus* | Delaware Bay, NJ, USA | 39 | -75 | 104 | 0 |  |
| 2015 | Pre-breeding | red knot | *Calidris canutus* | Padre Island, TX, USA | 27 | -97 | 11 | 0 |  |
| 2015 | Post-breeding | red knot | *Calidris canutus* | James Bay south, ON, Canada | 51 | -80 | 0 | 6 |  |
| 2015 | Post-breeding | red knot | *Calidris canutus* | Monomoy, MA, USA | 42 | -70 | 21 | 18 |  |
| 2015 | Post-breeding | red knot | *Calidris canutus* | Mingan Archipelago, QC, Canada | 50 | -64 | 59 | 60 |  |
| 2016 | Pre-breeding | red knot | *Calidris canutus* | Delaware Bay, NJ, USA | 39 | -75 | 110 | 0 |  |
| 2016 | Post-breeding | red knot | *Calidris canutus* | Delaware Bay, NJ, USA | 39 | -75 | 59 | 6 |  |
| 2016 | Post-breeding | red knot | *Calidris canutus* | James Bay south, ON, Canada | 52 | -81 | 8 | 1 |  |
| 2016 | Post-breeding | red knot | *Calidris canutus* | Monomoy, MA, USA | 42 | -70 | 88 | 10 |  |
| 2016 | Post-breeding | red knot | *Calidris canutus* | Mingan Archipelago, QC, Canada | 50 | -64 | 48 | 200 |  |
| 2014 | Post-breeding | ruddy turnstone | *Arenaria interpres* | Mingan Archipelago, QC, Canada | 50 | -64 | 0 | 1 |  |
| 2014 | Post-breeding | ruddy turnstone | *Arenaria interpres* | East Bay Island, NU, Canada | 64 | -82 | 1 | 0 |  |
| 2015 | Pre-breeding | ruddy turnstone | *Arenaria interpres* | Delaware Bay, NJ, USA | 39 | -75 | 46 | 0 |  |
| 2015 | Post-breeding | ruddy turnstone | *Arenaria interpres* | Coats Island, NU, Canada | 63 | -82 | 3 | 0 |  |
| 2015 | Post-breeding | ruddy turnstone | *Arenaria interpres* | East Bay Island, NU, Canada | 64 | -82 | 3 | 0 |  |
| 2016 | Pre-breeding | ruddy turnstone | *Arenaria interpres* | Delaware Bay, NJ, USA | 39 | -75 | 84 | 0 |  |
| 2016 | Post-breeding | ruddy turnstone | *Arenaria interpres* | Delaware Bay, NJ,USA | 39 | -75 | 2 | 0 |  |
| 2016 | Post-breeding | ruddy turnstone | *Arenaria interpres* | James Bay south, ON, Canada | 52 | -81 | 2 | 2 |  |
| 2016 | Post-breeding | ruddy turnstone | *Arenaria interpres* | Monomoy, MA, USA | 42 | -70 | 7 | 0 |  |
| 2015 | Pre-breeding | sanderling | *Calidris alba* | Chaplin, SK, Canada | 50 | -107 | 38 | 0 |  |
| 2015 | Pre-breeding | sanderling | *Calidris alba* | Padre Island, TX, USA | 27 | -97 | 24 | 0 |  |
| 2015 | Post-breeding | sanderling | *Calidris alba* | Bathurst Island, NU, Canada | 76 | -98 | 5 | 0 |  |
| 2015 | Post-breeding | sanderling | *Calidris alba* | James Bay south, ON, Canada | 52 | -81 | 0 | 1 |  |
| 2015 | Post-breeding | sanderling | *Calidris alba* | Gulf of Maine, ME, USA | 44 | -70 | 0 | 6 |  |
| 2016 | Pre-breeding | sanderling | *Calidris alba* | Padre Island, TX, USA | 27 | -97 | 38 | 0 |  |
| 2016 | Pre-breeding | sanderling | *Calidris alba* | Delaware Bay, NJ, USA | 39 | -75 | 50 | 0 |  |
| 2014 | Post-breeding | semipalmated sandpiper | *Calidris pusilla* | Chignecto Bay, NB, Canada | 45 | -65 | 35 | 0 |  |
| 2014 | Post-breeding | semipalmated sandpiper | *Calidris pusilla* | Minas Basin, NS, Canada | 45 | -64 | 60 | 10 |  |
| 2014 | Post-breeding | semipalmated sandpiper | *Calidris pusilla* | Gulf of Maine, ME, USA | 45 | -68 | 19 | 22 |  |
| 2014 | Post-breeding | semipalmated sandpiper | *Calidris pusilla* | Coats Island, NU, Canada | 63 | -82 | 29 | 0 |  |
| 2014 | Post-breeding | semipalmated sandpiper | *Calidris pusilla* | James Bay south, ON, Canada | 52 | -81 | 68 | 14 |  |
| 2014 | Post-breeding | semipalmated sandpiper | *Calidris pusilla* | Gulf of Maine, ME, USA | 43 | -71 | 19 | 11 |  |
| 2014 | Post-breeding | semipalmated sandpiper | *Calidris pusilla* | Mingan Archipelago, QC, Canada | 50 | -64 | 0 | 23 |  |
| 2014 | Post-breeding | semipalmated sandpiper | *Calidris pusilla* | Kamouraska, St. Lawrence, QC, Canada | 48 | -69 | 0 | 2 |  |
| 2015 | Pre-breeding | semipalmated sandpiper | *Calidris pusilla* | Delaware Bay, NJ, USA | 39 | -75 | 56 | 0 |  |
| 2015 | Post-breeding | semipalmated sandpiper | *Calidris pusilla* | Coats Island, NU, Canada | 63 | -82 | 18 | 0 |  |
| 2015 | Post-breeding | semipalmated sandpiper | *Calidris pusilla* | James Bay south, ON, Canada | 52 | -81 | 42 | 38 |  |
| 2015 | Post-breeding | semipalmated sandpiper | *Calidris pusilla* | Gulf of Maine, ME, USA | 43 | -71 | 59 | 19 |  |
| 2015 | Post-breeding | semipalmated sandpiper | *Calidris pusilla* | Kamouraska, St. Lawrence, QC, Canada | 48 | -69 | 12 | 0 |  |
| 2015 | Post-breeding | semipalmated sandpiper | *Calidris pusilla* | Mingan Archipelago, QC, Canada | 50 | -64 | 3 | 12 |  |
| 2016 | Post-breeding | semipalmated sandpiper | *Calidris pusilla* | James Bay south, ON, Canada | 52 | -81 | 10 | 26 |  |
| 2016 | Post-breeding | semipalmated sandpiper | *Calidris pusilla* | Mingan Archipelago, QC, Canada | 50 | -64 | 43 | 5 |  |
| 2016 | Post-breeding | semipalmated sandpiper | *Calidris pusilla* | Gulf of Maine, ME, USA | 44 | -70 | 20 | 8 |  |
|  |  |  |  |  |  |  |  |  |  |
|  |  |  |  |  |  |  |  |  |  |
|  |  |  |  |  |  |  |  |  |  |

**Table S2.** Flight cost calculations from *Flight* 1.24 for mean airspeeds per season. For specific parameters see Table S3.

**Species Airspeed (m s^-1^) Specific power (W kg^-1^)**

Season Pre-breeding Post-breeding Pre-breeding Post-breeding

red knot 12.0 13.9 41.6 45.8

ruddy turnstone 12.0 13.7 44.1 48.2

sanderling 10.5 13.1 44.4 50.3

semipalmated sandpiper 11.3 11.7 29.7 31.0

**Table S3.** Model parameters for *Flight* 1.24, where the induced power factor (*k*) was set at 0.9 the body drag coefficient (*C*_db_) at 0.1, and the minimum-power speed (*V*_mp_) and maximum-range speed (*V*_mr_) were calculated for sea-level air density.

**Species body mass (kg) Wing span (m) Wing area (m^2^) *V*_mp_ (m s^-1^) *V*_mr_ (m s^-1^)**

red knot 0.180 0.538 0.0332 10.1 16.7

ruddy turnstone 0.122 0.465 0.02379 9.6 15.9

sanderling 0.065 0.375 0.01548 9.3 15.4

semipalmated 0.027 0.346 0.01317 7.2 12.7

sandpiper

**
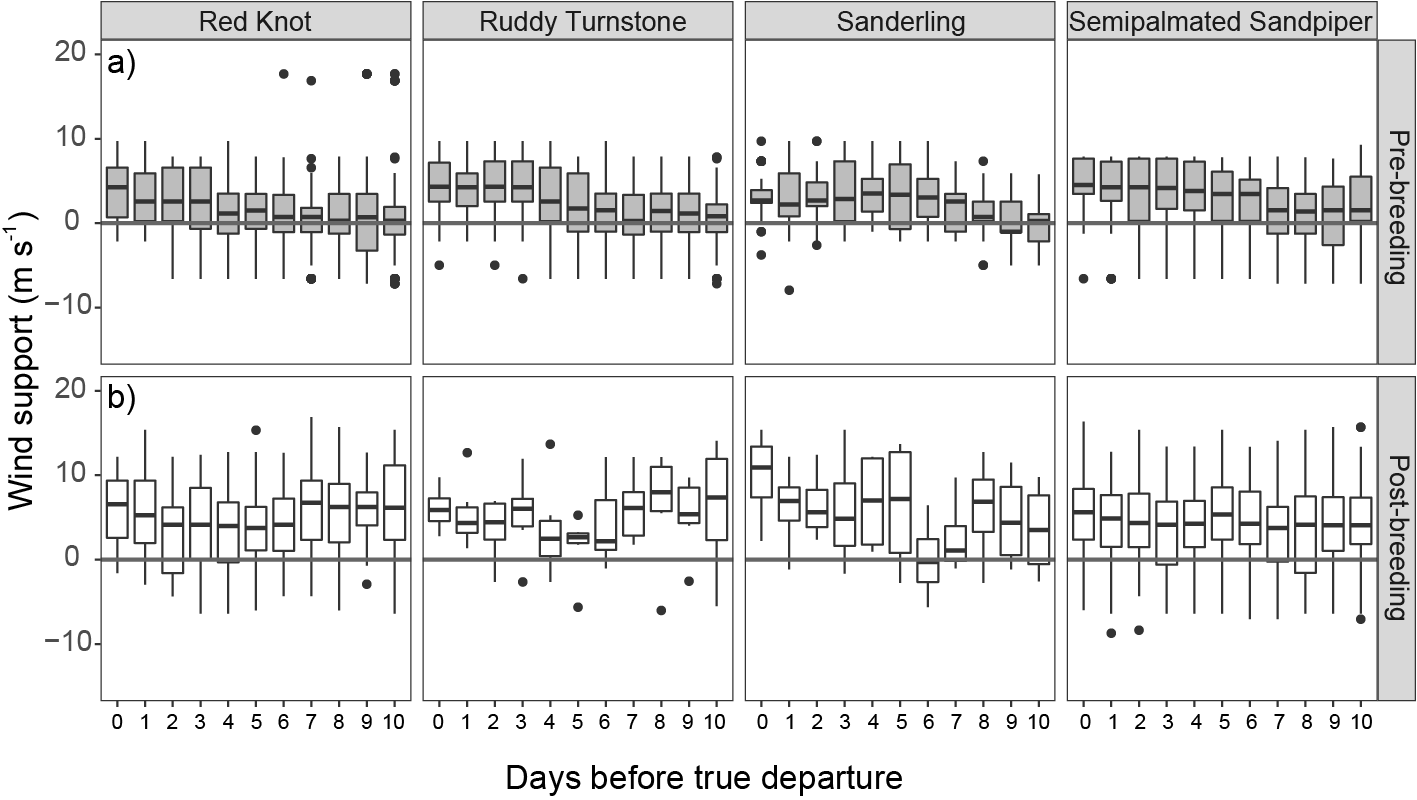
**

**Figure S1.** Boxplots of wind support that the four shorebird species would experience at different departure times from stopovers during (a) the pre-breeding season and (b) the post-breeding season. The horizontal line indicates whether birds experience tailwinds (> 0) or headwinds (< 0). Wind support at observed departure (day 0) is higher than up to 10 days before true departure in both seasons.
